# Vincristine-Induced Peripheral Neuropathy in Childhood Acute Lymphoblastic Leukemia: Genetic Variation as a Potential Risk Factor

**DOI:** 10.3389/fphar.2021.771487

**Published:** 2021-12-09

**Authors:** Qing-Yan Yang, Ya-Hui Hu, Hong-Li Guo, Ying Xia, Yong Zhang, Wei-Rong Fang, Yun-Man Li, Jing Xu, Feng Chen, Yong-Ren Wang, Teng-Fei Wang

**Affiliations:** ^1^ Pharmaceutical Sciences Research Center, Department of Pharmacy, Children’s Hospital of Nanjing Medical University, Nanjing, China; ^2^ School of Basic Medical Sciences and Clinical Pharmacy, China Pharmaceutical University, Nanjing, China; ^3^ Department of Hematology, Children’s Hospital of Nanjing Medical University, Nanjing, China; ^4^ Department of Pharmacology, Addiction Science and Toxicology, University of Tennessee Health Science Center, Memphis, TN, United States

**Keywords:** vincristine, VCR- induced peripheral neuropathy, pharmacogenomics, CEP72, polymorphism

## Abstract

Vincristine (VCR) is the first-line chemotherapeutic medication often co-administered with other drugs to treat childhood acute lymphoblastic leukemia. Dose-dependent neurotoxicity is the main factor restricting VCR’s clinical application. VCR-induced peripheral neuropathy (VIPN) sometimes results in dose reduction or omission, leading to clinical complications or affecting the patient’s quality of life. With regard to the genetic basis of drug responses, preemptive pharmacogenomic testing and simultaneous blood level monitoring could be helpful for the transformation of various findings into individualized therapies. In this review, we discussed the potential associations between genetic variants in genes contributing to the pharmacokinetics/pharmacodynamics of VCR and VIPN incidence and severity in patients with acute lymphoblastic leukemia. Of note, genetic variants in the *CEP72* gene have great potential to be translated into clinical practice. Such a genetic biomarker may help clinicians diagnose VIPN earlier. Besides, genetic variants in other genes, such as *CYP3A5, ABCB1, ABCC1, ABCC2, TTPA, ACTG1, CAPG, SYNE2, SLC5A7, COCH,* and *MRPL47,* have been reported to be associated with the VIPN, but more evidence is needed to validate the findings in the future. In fact, a variety of complex factors jointly determine the VIPN. In implementing precision medicine, the combination of genetic, environmental, and personal variables, along with therapeutic drug monitoring, will allow for a better understanding of the mechanisms of VIPN, improving the effectiveness of VCR treatment, reducing adverse reactions, and improving patients’ quality of life.

## 1 Introduction

Leukemia, the most common childhood malignant disease, is a group of diseases consisting of acute lymphocytic leukemia (ALL), chronic myeloid leukemia, and acute myeloid leukemia with large differences in morphology and cytogenetics. ALL is with the highest incidence, accounting for about 25% of childhood malignancies and 72.4% of childhood leukemia. In United States, the American Cancer Society’s estimate of ALL (both children and adults) in 2021 is approximately 5,690 new cases and 1,580 deaths, respectively. The global cure rate of ALL exceeds 85% (Pui and Evans, 2006; Pui et al., 2008). In China, according to China’s National Children’s Tumor Surveillance Annual Report 2020, leukemia is the most prevalent disease, accounting for 57.2% of discharged patients with cancer (Yi, 2021). The 5 years overall survival rate (OS) is 91% and event-free survival rate (EFS) is 81%, in accordance with the report of Acute Lymphoblastic Leukemia Clinical Research Program (CCCG-2015-ALL) in 2015 initiated by the Chinese Pediatric Oncology Committee (Tang, 2020).

Combination chemotherapy is the most important treatment strategy for childhood ALL patients in the phases of induction, consolidation/enhancement, and maintenance. Commonly used medications include cyclophosphamide (CTX), vincristine (VCR), daunorubicin (DNR), L-asparaginase (L-Asp), prednisone (PDN), and dexamethasone (DXM). Among them, VCR is the first-line medication to treat childhood ALL (Pui and Evans, 2006). This periwinkle alkaloid is also used for the treatment of solid tumors and other hematologic malignancies besides leukemia, including breast cancer and non-Hodgkin’s lymphoma (Below and Das, 2021). VCR interferes assemble of microtubules in the mitotic spindle causing mitosis disruption and cell death at metaphase (K. K. Gupta et al., 2006). Its main dose-limiting toxicity (Old et al., 2014) is VCR-induced peripheral neuropathy (VIPN), which is developed during treatment in approximately 80% of patients, affecting life quality even years post-treatment (Smith et al., 2015). Childhood exposure to VCR increased the risk of movement disorders during later life (Ness et al., 2013). Childhood ALL survivors who received chemotherapy were also hospitalized significantly more often than their siblings and the general population (Ou et al., 2017).

To prevent and reduce the occurrence of VIPN, individualized medical treatment is required, because the development of VIPN may involve multiple individualized risk factors, such as patient-related [i.e., age (Ceppi et al., 2014; Hershman et al., 2016), gender (Diouf et al., 2015), race (Sims 2016), genetic polymorphisms (Pozzi et al., 2021)] treatment-related risk factors [i.e., dosage (Okada et al., 2014), administration method (Qweider et al., 2007), and drug interactions (Moriyama et al., 2012)].

Nowadays, cancer therapy is shifting from the traditional “one-size-fits-all” to an individualized, specifically genetic defects-based approach, through which each patient will be diagnosed, treated, and monitored for interactions with drugs (S. Gupta et al., 2016). (Kamel and Al-Amodi, 2017). In particular, pharmacogenomic or pharmacogenetic studies reveal how a person’s unique genome influences his/her response to therapies. Preemptive pharmacogenetic testing is helpful for clinicians to tailor the dose of a drug for each individual patient so as to exert the best therapeutic effects and minimize potential adverse reactions in clinical practice. Further research established the pharmacogenomic approach as a powerful method for elucidating genetic variants for the classification of patients at low or high risk of developing chemotherapy–induced peripheral neuropathy (Q. Li et al., 2012). For this purpose, this review summarizes the genetic variants that have been studied related to VIPN in order to guide clinicians to individualize treatment.

## 2 VCR and VIPN

### 2.1 Pharmacokinetics of VCR

**FIGURE 1:**
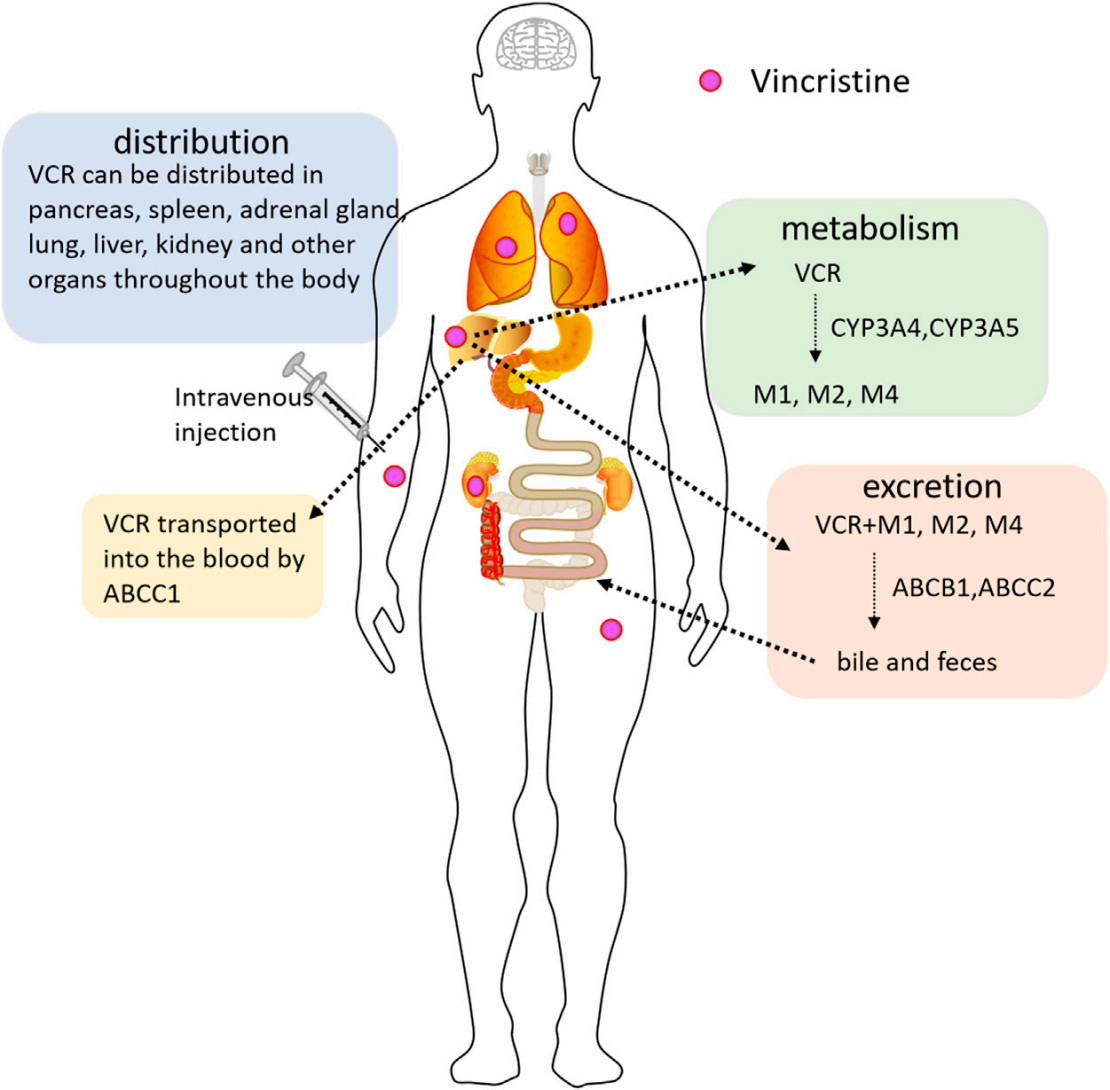
The pharmacokinetic pathway of VCR (Vincristine) in the body. VCR is mainly metabolized by the hepatocytic CYP3A4 and CYP3A5 enzymes, forming metabolites M1, M2, and M4. VCR is transported into the blood by ABCC1. The biliary excretion of VCR is mediated by ABCB1 and ABCC2. VCR is mainly excreted through bile and feces, and less through the kidneys.

Bi-exponential or tri-exponential kinetics is the characteristic of VCR plasma concentration-time curves. The VCR has a short distribution half-life, a long half-life, and a large volume of distribution at steady state, showing extensive tissue binding capacity (Gidding et al., 1999). Wide interpatient variability of the above indexes has been documented. VCR can be distributed and accumulated very high in multiple tissues, such as the pancreas, liver, intestinal mucosa, spleen, bone marrow, thyroid, adrenal gland, kidney, and lung. These pharmacokinetic manifestations limit the clinical efficacy of VCR due to its high binding capacity to normal tissue and limited tumor tissue exposure (Said and Tsimberidou, 2014). VCR is primarily metabolized in the liver by the CYP3A subtype, specifically CYP3A4 and CYP3A5 enzymes (Lee et al., 2019). M1 is the major metabolite. M2 and M4 are the two minor metabolites of VCR (Pozzi et al., 2021). In the human body, most VCR is excreted through bile and feces, and less through the kidneys (Bender et al., 1977; Lee et al., 2019). VCR is excreted as an unchanged parent drug along with metabolites (Jackson et al., 1978) (Figure 1). Transport and elimination of VCR are mediated by the membrane transport proteins, including ABCB1, ABCC1, ABCC2, etc. For example, excretion of VCR into bile is mediated by ABCB1 and ABCC2, versus into the blood by ABCC1 (Huang et al., 2006; Zgheib et al., 2018). Therefore, activity or expression of genes involved in the pharmacokinetics of VCR can change the exposure and drug effects of VCR, thereby affecting its toxicity.

### 2.2 Adverse Reactions to VCR

The major side effect of VCR is neurotoxicity, a dose-limiting peripheral neuropathy (Jain et al., 2014), which involves a wide range of dysfunction and is mainly categorized into sensory, motor and autonomic neuropathy. Patients with sensory neuropathy have sensory nerve damage with symptoms of symmetry sensory/tactile dysfunction, numbness and tingling sensation in both hands and feet (Balayssac et al., 2011; Schouten et al., 2020). Motor neuropathy is usually characterized by peripheral sensory numbness, paresthesia, impaired balance, tendon weakness, and gait changes (Kavcic et al., 2017). Patients with autonomic neuropathy have dysuria, erectile hypotension, sexual dysfunction, and paralytic ileus (Balayssac et al., 2011). In addition, VCR can cause cranial neuropathy, resulting in vision and hearing dysfunction even blindness and deafness (Nazir et al., 2017).

Hematological toxicity is one of the uncommon adverse reactions. Mild and recurrent myelosuppression in terms of leukopenia, anemia, and neutropenia (Johnson et al., 1963; Niu et al., 2018; Joseph et al., 2020) in addition to severe myelosuppression and thrombocytosis may occur (Carbone et al., 1963; Kaufman et al., 1976). Different degrees of alopecia may also develop (Gidding et al., 1999), and the reported incidence associated with VCR ranges from 8 to 23.1% (Niu et al., 2018; Joseph et al., 2020). Other rare side effects associated with VCR include fever, gastrointestinal ulcers and perforation, myocardial infarction, rashes (Niu et al., 2018; Joseph et al., 2020).

### 2.3 Pathogenesis of VIPN

**FIGURE 2:**
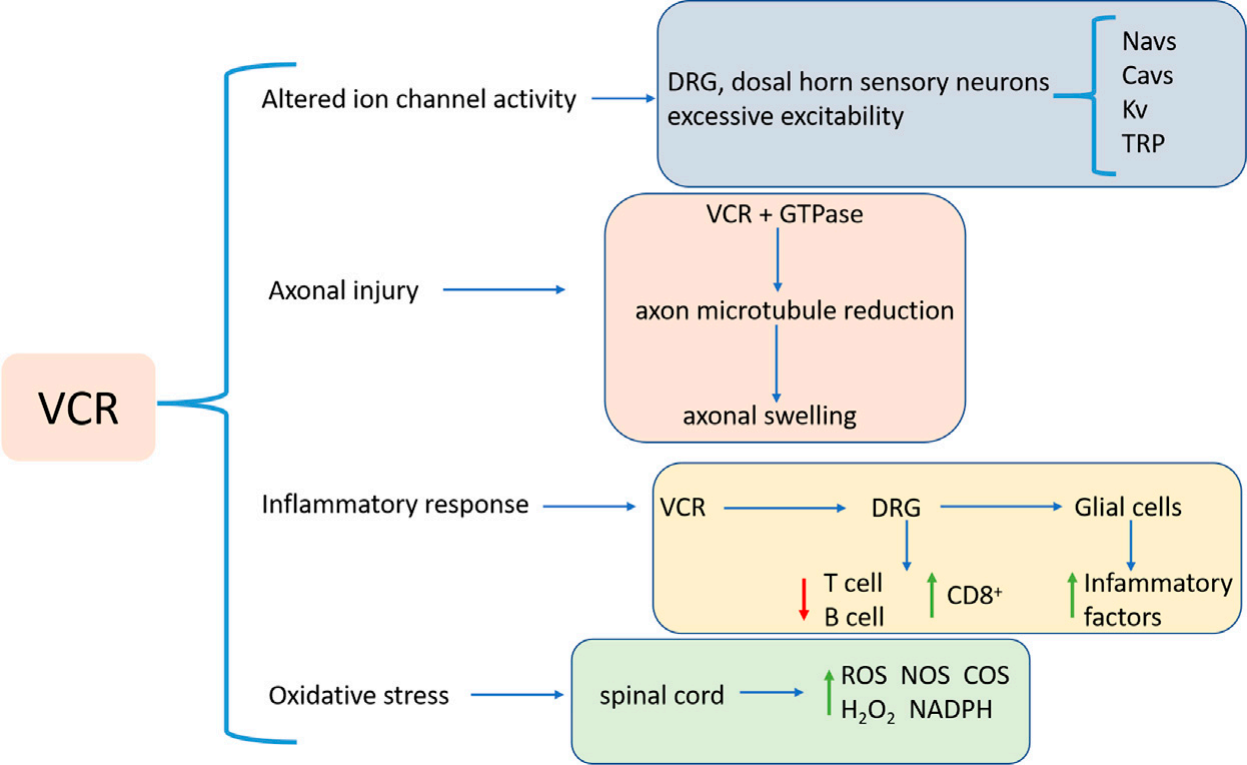
VIPN pathogenesis, which involves axonal injury, altered ion channel activity, inflammatory response, and oxidative stress. Abbreviations: VCR: Vincristine; DRG: dorsal root ganglion; CD8+: cytotoxic T cells; ROS: Reactive oxygen species; NOS: nitric oxide synthase; COS: cyclooxygenase; NADPH: nicotinamide adenine dinucleotide phosphate; Navs: Voltage-gated sodium channels; Cavs: Voltage-gated calcium channels; Kv: Voltage-gated potassium; TRP: transient receptor potential.

The mechanism of VIPN may be at least partially related to axonal injury, altered ion channel activity, inflammatory response, and oxidative stress (Figure 2).

Firstly, the development of VIPN is associated with the effect of VCR on microtubules. Induced by VCR a stable complex in the tubulin GTPase domain is formed, which inhibits GTP hydrolysis and polymerization of soluble dimers to form microtubules (Argyriou et al., 2012), causing loss of axonal microtubules and alteration of the length, arrangement and orientation, consequentially leading to swelling of axons in both myelinated and unmyelinated fibers (Topp et al., 2000). VCR also interferes microtubule functions, blocks the axonal conduction, resulting in axonal degeneration, and subsequent demyelination (Bradley et al., 1970; Callizot et al., 2008).

The second factor is ion channel activity. The activity of different ion channels leads to hyper-excitability and adverse reactions of sensory neurons in the plasma membrane of the dorsal root ganglion (DRG) and sensory neurons in the dorsal horn of patients. Voltage-gated sodium channels (Navs) primarily generate excitatory action potentials. Nav subunits are involved in peripheral pain processing and increased sensitivity to pain (Lampert et al., 2010; Emery et al., 2016). The particular Nav subunits associated with VIPN are Nav1.3, Nav1.6, Nav1.7 and Nav1.8 (Joshi et al., 2006; Üçeyler et al., 2006; Lampert et al., 2010). Voltage-gated calcium channels (Cavs) are also involved in neuronal excitability. VCR treatment results in intracellular Ca^2+^ imbalance, leading to mechanical tenderness and mechanical hyperalgesia. Cav3.2 and CaMKII-mediated Cx43-dependent inflammations play a role in VCR-induced neuropathic pain (G.-Z. Li et al., 2021). Pathologic pain caused by VCR could be significantly alleviated by inhibiting Cav3.2 T-type calcium channels (Jarvis et al., 2014; Sharma et al., 2020). Potassium channels (Kv) are associated with VIPN onset as well. Kv channel has a critical physiological role in regulating membrane potential in excitable tissues such as pain-sensing neurons (Takeda et al., 2011). Chemical and physical sensors that act as signals of thermal, chemical, and mechanical stimuli cause pathologic pain (Dai, 2016; Laing and Dhaka, 2016). Transient receptor potential (TRP) channels, as molecular detectors of chemical and physical stimuli, are expressed in peripheral sensory neurons, including DRG neurons (Salzer et al., 2019) and have been shown to play a role in sensory neuron-mediated pain (Sikand and Premkumar, 2007; Carrasco et al., 2018).

Thirdly, inflammation is associated with the progression of VIPN. Chemotherapeutic drugs can reduce anti-inflammatory T cells and B cells and increase cytotoxic T cells (CD8^+^) by triggering an adaptive immune response in DRG neurons (Liu et al., 2014; Krukowski et al., 2016). Moreover, glial cells, e.g., microglia and astrocytes, activated by peripheral nerve injury can produce and secrete pro-inflammatory factors (Shamash et al., 2002; Shen et al., 2015; Zhou et al., 2018). Up-regulating pro-inflammatory cytokines then leads to inflammation and deleterious neuronal sensitization (Kiguchi et al., 2008; Ji et al., 2013), while down-regulation of anti-inflammatory cytokines may undermine the inhibitory effect on inflammation (Nie et al., 2017).

Lastly, VCR is also involved in the body’s oxidative stress response. Studies have shown that VCR increased ROS production, H_2_O_2_ levels and nicotinamide adenine dinucleotide phosphate (NADPH) oxidase activity in mouse spinal cord samples (Chen et al., 2020). The production of cyclooxygenase (COS) and nitric oxide synthase (NOS) promotes the transmission of pain signals in VIPN (Singh et al., 2019). Moreover, activation of the L-arginine/NO/cGMP pathway is associated with VCR-induced hyperalgesia (Kamei et al., 2005).

### 2.4 Risk Factors for VIPN

Several factors may affect the incidence and severity of VIPN. First is the dosage regimen. In general, a single dose of VCR is 1.4–1.6 mg/m^2^ (Okada et al., 2014). A previous study confirmed an increased risk of early-onset VIPN in patients receiving an initial dose of at least 1.9 mg (Okada et al., 2014). When the total cumulative dose of VCR was 30–50 mg, nearly 60% of patients developed neuropathy, mainly primary sensory or sensorimotor neuropathy (Argyriou et al., 2014). However, the studies investigating the effect of dose on the development or severity of VIPN provided inconclusive results (Velde et al., 2017). Moreover, some researchers suggest that a positive correlation exists between neuropathy and peak plasma concentrations of VCR, although data supportive of this hypothesis do not exist (Said and Tsimberidou, 2014). The combination of VCR and azole antifungal agents also affects the VIPN symptoms (Moriyama et al., 2012).

Age, gender, physiological conditions, and comorbidity also have an impact on VIPN. Studies have shown that for every additional year of age, the risk of neuropathy increases by 4% (Hershman et al., 2016). But contradictory results regarding the relationship between VIPN and age at study have been reported (Velde et al., 2017). Toopchizadeha and others have shown that women have a higher incidence of VIPN (Barzegar et al., 2015), whereas other study found boys to be at higher risk or did not find a significant relation between sex and VIPN in children (Velde et al., 2017). Patients with obesity, diabetes, Charcott-Marie-tooth and obstructive liver disease develop severe VIPN after treatment with VCR (Gidding et al., 1999; Emery et al., 2016; Hershman et al., 2016; Sajdyk et al., 2020). In contrast, co-morbidity of autoimmune diseases was associated with a reduced risk of VIPN (Hershman et al., 2016).

The severity of VIPN varies among ethnic groups. The risk and severity of VIPN in whites are higher than that in African Americans, but the survival rate is higher than that in African Americans (Pollock et al., 2000; Sims, 2016).

In addition to the above risk factors, particular genetic variants are a critical factor that has attracted more attention. Of note, associations between genetic variants and VIPN have been identified in various studies.

## 3 Pharmacogenomics Relevant to VCR

**FIGURE 3:**
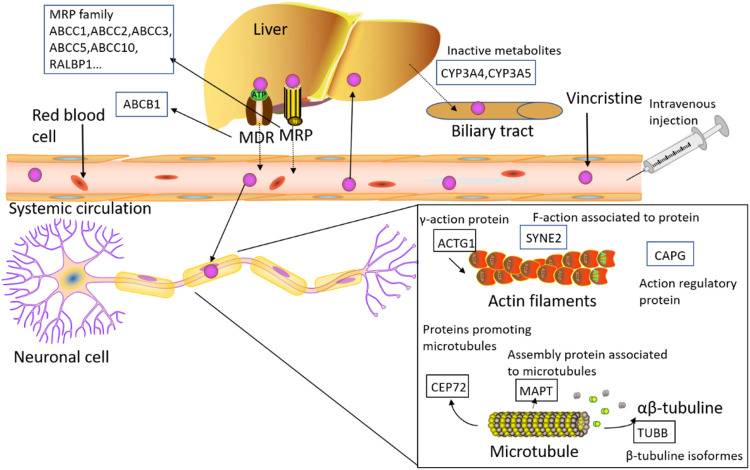
The figure shows the detected genes involved in the PK/PD process of VCR, including *ABC* transporters, *CEP72* (centrosomal protein 72), *ACTG1* (actin gamma 1), *CAPG* (capping actin protein), *SYNE2* (spectrin repeat containing nuclear envelope protein 2), *MAPs* (microtubule-associated proteins), *TUBB* (beta tubulin), *RALBP1* (rala binding protein 1), *ABCB1* (ATP-binding cassette subfamily B member 1), *ABCC1* (ATP-binding cassette subfamily C member 1), *ABCC2* (ATP-binding cassette subfamily C member 1), *ABCC3* (ATP-binding cassette subfamily C member 3), *ABCC5* (ATP-binding cassette subfamily C member 5) and *ABCC10* (ATP-binding cassette subfamily C member 10).

In the present review, we focused on the genetic variability associated with the risk of developing VIPN and we discussed different genes and genetic variants that could be involved in the pharmacokinetics and/or pharmacodynamics of VCR, which might determine the course and severity of VIPN. Commonly evaluated genetic variants include single-nucleotide polymorphisms (SNPs), genomic insertions and deletions, and genetic copy number variations. SNPs are the most frequent of the inherited sequence variations. Here we discuss past and current reports in pharmacogenomic studies regarding VIPN and the extent to which findings have the potential to have an impact on cancer patient care (Table 1; Figure 3).

**TABLE 1:** Pharmacogenomic studies of VIPN.

**Gene**	**SNP reference No.**	**GRCh38.p13**	**Research association with VIPN**			**Research non-association with VIPN**
			**Research results**	**Total number of patients participating in the trial**	**References**	
Genes related to metabolism of VCR						
CYP3A5*1	CYP3A5*1*3(CYP3A5 expressers)		↓ Risk and severity of VIPN	107 children with ALL	Egbelakin et al. (2011)	Aplenc et al. (2003); Hartman et al. (2010); Guilhaumou et al. (2011); Moore et al. (2011); Ceppi et al. (2014); Franca et al. (2017); Skiles et al. (2018)
	CYP3A5*3*3 (CYP3A5 non-expressers)		↑ Risk and severity of VIPN	107 children with ALL	Egbelakin et al. (2011)	Aplenc et al. (2003); Hartman et al. (2010); Guilhaumou et al. (2011); Moore et al. (2011); Ceppi et al. (2014); Franca et al. (2017); Skiles et al. (2018)
CYP3A5*3	rs776746	g.99672916T > C	↑ Risk and severity of VIPN (*p* = 0.021)	533 children with ALL	(Aplenc et al. (2003); Sims (2016)	Hartman et al. (2010); Guilhaumou et al. (2011); Moore et al. (2011); Ceppi et al. (2014); Franca et al. (2017); Skiles et al. (2018)
				53 children with ALL		
CYP3A5*6	rs10264272	g.99665212C > T		No association		Moore et al. (2011) Aplenc et al. (2003); Hartman et al. (2010); Guilhaumou et al. (2011); Ceppi et al. (2014); Franca et al. (2017); Skiles et al. (2018)
CYP3A4*1B	rs2740574	g.99784473C > T/A/G	↓ Risk and severity of VIPN (*p* = 0.024)	533 children with ALL	Aplenc et al. (2003)	Guilhaumou et al. (2011)
Genes related to transport of VCR						
*ABCB1*	rs1045642	g.87509329A > T/A/G	↑ Risk and severity of VIPN	339 children with ALL	Jamroziak et al. (2004); Stanulla et al. (2005); Ceppi et al. (2014); Zgheib et al. (2018)	Plasschaert et al. (2004); Hartman et al. (2010)
				113 children with ALL		
				68 children with ALL		
				133 children with ALL		
*ABCB1*	rs4728709	g.87604286G > A	↓ Risk of low-grade VIPN	339 children with ALL	Ceppi et al. (2014)	—
*ABCB1*	rs1128503	g.87550285A > G		No association		Ceppi et al. (2014)
*ABCB1*	rs10244266	g.87559151T > G	↑ Risk and severity of grade 1–2 VIPN (*p* = 0.005)	152 children with ALL	Lopez-Lopez et al. (2016) (grade1-2)	Lopez-Lopez et al. (2016) (grade 3–4)
*ABCB1*	rs10274587	g.87535167G > A	↑ Risk and severity of grade 1–2 VIPN (*p* = 0.009)	152 children with ALL	Lopez-Lopez et al. (2016) (grade1-2)	Lopez-Lopez et al. (2016) (grade 3–4)
*ABCB1*	rs10268314	g.87540353T > C	↑ Risk and severity of grade 1–2 VIPN (*p* = 0.018)	152 children with ALL	Lopez-Lopez et al. (2016) (grade1-2)	Lopez-Lopez et al. (2016) (grade 3–4)
*ABCB1*	rs2032582	g.87531302A > T/C	No association			Plasschaert et al. (2004); Hartman et al. (2010)
*ABCB1*	—	g.G1199A	Affected VCR outflow from cells	—	Woodahl et al. (2009)	—
*ABCB1*	—	g.G554T	Changed the pharmacological properties of VCR	—	Choi et al. (1988); Wolf et al. (2011)	—
*ABCC1*	rs3784867	g.16109488C > T	↑ VIPN severity grade (*p* = 5.34 × 10–5, OR = 4.91 95% CI: 1.99–12.10)	167 children with ALL	Wright et al. (2019)	—
*ABCC1*	rs3887412	g.16081173A > T	↑ Frequency episodes of delayed neurotoxicity	833 patients with multiple myeloma	Broyl et al. (2010)	—
*ABCC1*	rs246240	g.16025167A > T/G	↑ Frequency episodes of 3–4 grade neurotoxicity (OR: 4.61; 95% CI: 1.12–19.02)	311 children with ALL	Franca et al. (2017)	—
*ABCC1*	rs1967120	g.16015037G > C/A	↑ Frequency episodes of 3–4 grade neurotoxicity (*p* = 0.035)	152 children with ALL	Lopez-Lopez et al. (2016)	—
*ABCC1*	rs3743527	g.16141824C > T	↑ Risk and severity of VIPN	235 children with ALL	Semsei et al. (2012); Lopez-Lopez et al. (2016)	—
				152 children with ALL		
*ABCC2*	rs3740066	g.99844450C > T/G	↑ Risk and severity of grade 1–2 VIPN (*p* = 0.0002)	152 children with ALL	Lopez-Lopez et al. (2016)	—
*ABCC2*	rs12826	g.99852563C > T/A	↑ Risk and severity of grade 1–2 VIPN (*p* = 0.0001)	152 children with ALL	Lopez-Lopez et al. (2016)	—
*ABCC2*	rs2073337	g.99807669A > T/G		No association		Lopez-Lopez et al. (2016)
*ABCC2*	rs4148396	g.99832187T > C		No association		Lopez-Lopez et al. (2016)
*ABCC2*	rs11192298	g.105047053T > G		No association		Lopez-Lopez et al. (2016)
*ABCC2*	rs717620	g.99782821C > T		No association		Zgheib et al. (2018)
*ABCC3*	—	—		No association		Lopez-Lopez et al. (2016)
*ABCC10*	—	—		No association		Lopez-Lopez et al. (2016)
*SLC5A7*	rs1013940	g.107992192A > G	↑ Risk and severity of VIPN (*p* = 9.00 × 10–4, 8.60, 95%CI: 1.68–44.15)	167 children with ALL	Wright et al. (2019)	—
Genes related to target receptors of VCR						
*CEP72*	rs924607	g.609978C > T	↑ Risk and severity of VIPN (*p* = 9.00 × 10–4, 8.60, 95%CI: 1.68–44.15)	167 children with ALL	Diouf et al. (2015); Stock et al. (2017); Wright et al. (2019); Zečkanović et al. (2020)	A. Gutierrez-Camino et al. (2016); Zgheib et al. (2018)
				96 children with ALL		
				38 children with ALL		
				817 children with ALL		
*TUBB*	—	—		No association		Ceppi et al. (2014); Martin-Guerrero et al. (2019)
*ACTG1*	rs1135989	g.81510981G > C/A	↑ Risk and severity of grade 3–4 VIPN (OR: 2.8; 95% CI: 1.3–6.3; *p* = 0.008)	339 children with ALL	Ceppi et al. (2014)	—
*CAPG*	rs3770102	g.85410714G > T/A/C	↓ Risk and severity of grade 3–4 VIPN (OR:0.1; 95% C: 0.01–0.8; *p* = 0.009)	339 children with ALL	Ceppi et al. (2014)	—
*CAPG*	rs2229668	g.85401860C > T/G	↑ Risk and severity of grade 3–4 VIPN (OR: 2.1; 95% CI: 1.1–3.7; *p* = 0.02)	339 children with ALL	Ceppi et al. (2014)	—
*MAP4*	rs11268924; rs1137524; rs1875103	g.47914934C > G/T; g.47914934C > G/T; g.47852611C > T		No association		Ceppi et al. (2014)
*MAPT*	rs11867549	g.45935869A > G		No association		Hartman et al. (2010); Skiles et al. (2018); Martin-Guerrero et al. (2019)
Other genes						
*SYNE2*	rs2781377	g.64093374G > A	↑ Risk and severity of VIPN (OR: 2.5; 95% CI:1.2–5.2; *p* = 0.01)	237 children with ALL	Abaji et al. (2018)	—
*BAHD1*	rs3803357	g.40459356C > G/A	↓ Frequency episodes of 3–4 grade neurotoxicity (OR: 0.35; 95% CI: 0.2–0.7; *p* = 0.007)	237 children with ALL	Abaji et al. (2018)	—
*COCH*	rs1045644	g.30885890C > G	↓ Risk and severity of VIPN (*p* = 8.65E-07)	1696 children with B-ALL	[L. Li et al. (2019)]	—
*MRPL47*	rs10513762	g.179588987C > T		No association		Abaji et al. (2018)
*ITPA*	rs1127354	g.3213196C > G/A	↑ Frequency episodes of 3–4 grade neurotoxicity (OR: 13.23, 95% CI: 1.74–100.65, *p* = 0.013)	4 children with ALL	Stocco et al. (2009); Adam de Beaumais et al. (2011)	Hwang et al. (2012); Wan Rosalina et al. (2012)
				246 children with ALL		
*DPYD*	rs1413239	g.97221459C > T/A/G	↑ Risk and severity of delayed VIPN (*p* = 5.40 × 10-³)	833 patients (aged 18–65 years)	Broyl et al. (2010)	—
*RALBP1*	—	—		No association		Lopez-Lopez et al. (2016)
*TTPA*	rs10504361	g.62972541G > A	↑ Risk and severity of VIPN (*p* = 6.85 × 10–4, OR: 1.98 95%CI: 1.34–2.94)	167 children with ALL	Vainionpää et al. (1995); Wright et al. (2019)	—
				38 children with ALL		
*miR-3117-3p*	rs12402181	g.66628488G > T/A	↓ Risk and severity of VIPN (*p* = 0.00042)	179 children with B-ALL	(Á. Gutierrez-Camino et al. (2018)	—
*miR-4481*	rs7896283	g.12653178A > T/G		No association		(Á. Gutierrez-Camino et al. (2018)

Abbreviation: VCR: vincristine; VIPN: VCR-induced neuropathic pain; SNP, single nucleotide polymorphism; OR, odds ratio; CI, confidence interval; ALL, acute lymphoblastic leukemia; B-ALL, B-cell chronic lymphoblastic leukemia.

### 3.1 CYP450 Genes Involved in the Metabolism of VCR

VCR is catalyzed by the CYP3A enzyme. CYP3A5, not CYP3A4, is the functional enzyme that aids the liver in the clearance of VCR (Egbelakin et al., 2011). CYP3A4 and CYP3A5 both metabolize VCR to the major metabolite M1, but the M1 formation rate (Vmax) catalyzed by CYP3A5 is 7–9 times higher than that of CYP3A4, indicating that CYP3A5 is much more efficient at oxidizing VCR than CYP3A4 (Dennison et al., 2006). Thus, genetic polymorphisms in CYP3A5 expression may contribute to the interindividual variability in the metabolism of VCR, thereby altering the risk of VIPN. At least 17 different alleles (*1A, *1D, *2, *3A, *3B, *3D, *3F,*3G, *3J, *3K, *3L, *4, *5, *6, *7, *8, *9, *10, and *11) have been identified in the *CYP3A5* gene (https://www.pharmvar.org/gene/CYP3A5; Access time October 8, 2021), with *1 and *3 occurring frequently and being the most well studied (J.-Y. Sun et al., 2018). The CYP3A5*1 allele produces an active enzyme. CYP3A5*3, CYP3A5*6, and CYP3A5*7 mutations result in few or no functional enzymes. Intrinsic VCR clearance was 5 times higher in the CYP3A5*1 expression type than in the non-expression type (Dennison et al., 2007). The wild-type CYP3A5*1/*1 is uncommon in the Caucasians both in America and Europe (<1%), less common in East Asians (7%), intermediate in Hispanics (14%), but most common in African-Americans (37–45%) (J.-Y. Sun et al., 2018). Caucasians with the *CYP3A5* gene variant have increased susceptibility to mutations such as *3, *6, and *7. The CYP3A5*6 and CYP3A5*7 alleles result in nonfunctional truncated proteins. CYP3A5*3 is found in all ethnic populations and is the most common allele in Caucasians (Moore et al., 2011). CYP3A5*3 is the most common functionally missing variant located in intron 3, leading to premature termination of the codon (Egbelakin et al., 2011). *CYP3A5* expressers have a lower incidence of VIPN (*p* = 0.03), lower neurotoxicity grade and shorter duration of neurotoxicity (*p* = 0.035 and 0.0007) (Egbelakin et al., 2011).

Sims et al. reported that the CYP3A5*3 genotype leads to extremely low expression of CYP3A5 protein, which reduces VCR metabolism. Patients carrying CYP3A5*3 had a higher incidence of VIPN and, consistently, there was a higher incidence of neurotoxicity in Caucasian patients (Caucasian vs. African-American: 80.6% vs. 76.5%, *p* = 0.730) (Sims, 2016). Egbelakin et al. found that CYP3A5 *1*3 and *3*3 genotypes were associated with VCR exposure and VIPN phenotypes in a cohort of American subjects. When compared to *CYP3A5* non-expressers, *CYP3A5* expressers have higher level of M1 metabolite (M1) (*p* = 0.0004) and lower metabolic ratios ([VCR]/[M1]) (*p* = 0.036). M1 concentration was negatively correlated with the severity of neuropathy (*p* = 0.0316) (Egbelakin et al., 2011). Aplenc et al. found that CYP3A5*3 carriers had a decreased risk of VIPN that was statistically significant on univariate analysis, but not after controlling for multiple comparisons (Aplenc et al., 2003). This finding is in line with several other studies (Hartman et al., 2010; Moore et al., 2011; Guilhaumou et al., 2011; Ceppi et al., 2014; Franca et al., 2017). In these studies, there were associations between *CYP3A5* genotypes and VIPN development. Skiles et al. demonstrated that the dose and BSA normalized AUC of *CYP3A5* low-expressers were significantly higher than those of *CYP3A5* high-expressers (0.28 ± 0.15 h⋅m^2^/L vs. 0.15 ± 0.011 h⋅m^2^/L, *p* = 0.027). Moreover, the occurrence or severity of VIPN between the two groups was the same, which was assessed by a Total Neuropathy Scale tool (Skiles et al., 2018).

### 3.2 Genes Involved in the Transport of VCR

#### 3.2.1 ABC Transporter Genes

The ATP-binding cassette transporter (ABC) is an ancient and large family of transporters. As a kind of ATP-driven pump, the ABC membrane transporters consist of two transmembrane domains and two cytoplasmic ATP-binding domains. ABC membrane transporters have similar material transport functions and structures. But as the gene evolves, there are many differences between the members. ABC family transporters, including ABCB1, ABCC1, ABCC2, and ABCB4, play critical roles in VCR transport and clearance. Biliary excretion of VCR is mediated by ABCB1 and ABCC2, while VCR is transported into the blood by ABCC1 (Lopez-Lopez et al., 2016).

##### 3.2.1.1 ABCB1

*ABCB1* is an environmentally susceptible gene that codes for P-glycoprotein (P-gp; or multidrug resistance protein 1, MDR1). The P-gp transporter is located in the luminal membrane of the small intestine and blood-brain barrier, and in the apical membranes of excretory cells such as hepatocytes and kidney proximal tubule epithelia. P-gp transporter found on intestinal epithelial cells is responsible for efflux that limits cellular uptake and absorption into enterocytes. While P-gp transporter expressed on the canalicular surface of hepatocytes and renal tubular cells enhances elimination of drugs into the bile and urine (Wessler et al., 2013).

Cancer cells can become resistant to a broad spectrum of structurally and mechanistically distinct antitumor drugs with underlying molecular mechanisms, at least including diminished intracellular drug concentration via impaired drug influx and/or enhanced drug extrusion mediated by multidrug resistance (MDR) efflux transporters, and P-gp is the best characterized efflux pump known to play an important role in chemotherapeutic drug efflux in cancer cells (Dong et al., 2020). Recently, genetic polymorphisms of the *ABCB1* gene and their effects on the pharmacokinetics of various drugs, including VCR, have been elucidated. The *ABCB1* gene polymorphism (rs4728709; g.87604286G > A) may modulate VCR-associated Grade I/II neurotoxicity (Ceppi et al., 2014). In cellular experiments, the P-gp mutation (G554T; Gly185Val; rs1128501) reduced the resistance of cells to VCR (Safa et al., 1990).

Existing reports revealed that genetic variants (rs4728709, rs1045642, rs1128503, rs2032582, rs10244266, rs10274587, rs10268314, G119A and G554T) of the *ABCB1* gene may be related to VCR’s pharmacokinetics, pharmacodynamics, and adverse reactions. Patients with the ABCB1 T allele (rs4728709; g.87604286G > A) had a lower risk of toxicity (OR 0.3; 95% CI 0.1–0.9; *p* = 0.02), fewer toxicity episodes (*p* = 0.04), and tolerated higher VCR doses (*p* = 0.02). This C > T substitution in *ABCB1* had a protective effect against low grade I/II neurotoxicity (*p* = 0.01) (Ceppi et al., 2014). *ABCB1* rs1045642 (g.87509329A > T/A/G) and rs1128503 variants were in a state of linkage disequilibrium, and the Pearson correlation was 0.725 (*p* < 0.001). Rs1045642 is a common variant and is known to be associated with less-of-function protein expression, thereby predicting that rs1045642 T allele carriers have a poor ability to pump VCR out of the hepatocytes into the bile, leading to higher plasma VCR levels and hence higher toxicity (Zgheib et al., 2018). Other research has found that the ABCB1 variants rs10244266, rs10274587, and rs10268314 are associated with Grade I or II neurotoxicity but not with high toxicity (Grade III or IV) (Lopez-Lopez et al., 2016). Experiments revealed that patients with the *ABCB1* rs1045642 TT genotype had a lower event-free survival (Ceppi et al., 2014). It was found that CC genotype carriers (rs1045642) have a better event-free survival probability and a lower recurrence rate of peripheral adverse reactions in pediatric ALL patients (Jamroziak et al., 2004; Stanulla et al., 2005). But, this g.87509329A > T/A/G polymorphism was not associated with prognosis (Jamroziak et al., 2005). Plasschaert *et al.* performed *ABCB1* rs1045642 and rs2032582 (g.87531302A > T/C) variants genotyping in 52 Dutch childhood ALL patients, and the results showed no association between these two genotypes and the pharmacokinetics of VCR (Plasschaert et al., 2004). Similarly, genotyping of 36 Dutch children with ALL conducted by Hartman et al. showed that these polymorphisms exerted no effect on motor performance (Hartman et al., 2010). In addition, *in vitro* studies showed that G1199A (Ser400Asn) affected VCR efflux from cells (Woodahl et al., 2009), and G554T caused the substitution of amino acid Gly185Val and changed the pharmacological properties of anticancer drugs such as VCR (Choi et al., 1988; Wolf et al., 2011). The polymorphism of the *ABCB1* gene might also affect the pharmacokinetics and/or drug resistance of several other drugs in combination chemotherapy, thereby affecting the therapeutic effectiveness and adverse reactions.

##### 3.2.1.2 ABCC1

Multidrug resistance-associated protein 1 (MRP1) is a protein that is encoded by the *ABCC1* gene (also known as the *MRP1* gene) in humans. This protein functions as a multispecific organic anion transporter, with oxidized glutathione, cysteinyl leukotrienes, and activated aflatoxin B1 as substrates. This protein also transports glucuronides and sulfate conjugates of steroid hormones and bile salts. ABCC1 is highly expressed in the heart, lung, testis, kidney, and placenta (Semsei et al., 2012). ABCC1 participates in detoxification, protects cells from the toxic effects of foreign substances, and also participates in the defense mechanism against oxidative stress (Bakos and Homolya, 2007). Kerb et al. found that *ABCC1* gene polymorphism could affect the function of transporters (Kerb et al., 2001). In childhood T-lineage ALL (T-ALL) patients, multidrug resistance is usually mediated by ABC proteins, mainly ABCB1 and ABCC1 (Efferth et al., 2006). Winter *et al.* found that ABCC1 was significantly upregulated (nearly 30 times) in T-ALL cell line model under selective VCR exposure, demonstrating that *ABCC1* contributes to the resistance of T-ALL to VCR (Winter et al., 2013). They also confirmed that VCR-resistant cells could actively transported VCR outside of themselves, and VCR-resistant cells displayed increased resistance to several common chemotherapeutic agents such as daunorubicin and prednisone, but not to L-asparaginase, indicating multidrug resistance capacity (Winter et al., 2013). In addition, the *ABCC1* g.16109488C > T variant (rs3784867) increases the severity of VIPN (Wright et al., 2019).

Wright *et al.* showed that *ABCC1* rs3784867 was associated with VIPN (*p =* 5.34 × 10^−5^; OR 4.91; 95% CI 1.99–12.10, specificity = 0.88; sensitivity = 0.36) (Wright et al., 2019). VCR-induced delayed peripheral neuropathy was linked to the ABCC1 gene variant rs3887412 (OR 3.36; 95%CI 1.47–7.67; *p* = 5.70 × 10^−3^) (Broyl et al., 2010). Predicting the transcription factor binding site near rs3743527, so it may also participate in drug elimination through ABCC1 (Semsei et al., 2012; Lopez-Lopez et al., 2016). Lopez-Lopez et al. also found that SNP rs1967120 in the *ABCC1* gene was related to Grade III/IV VIPN, but this association was not statistically significant after false discovery rate (FDR) correction (Lopez-Lopez et al., 2016). As for homozygous SNP rs246240 (g.16025167A > T/G) in the *ABCC1* gene, minor allele G was associated with the onset of Grade III/IV neurological toxicity in the induction phase of childhood ALL patients with the AIEOP-BFM ALL 2000 study protocol (OR 4.61; 95% CI 1.12–19.02) (Franca et al., 2017).

##### 3.2.1.3 ABCC2 and Other ABCC Genes

Multidrug resistance-associated protein 2 (MRP2), also called canalicular multi-specific organic anion transporter 1 (cMOAT) or ATP-binding cassette sub-family C member 2 (ABCC2), is a protein encoded by the *ABCC2* gene in humans. ABCC2 plays a key role in the removal of various drugs and foreign biological agents from the biliary tract. Excessive protein may allow VCR to be transported out of cancer cells before VCR has its intended effect (Folmer et al., 2007). Lopez-Lopez et al. found SNPs rs2073337, rs4148396, and rs11192298 in the *ABCC2* gene have no significant association with the development of neurotoxicity, but *ABCC2* genotypes GG (rs3740066) and GG (rs12826) were associated with increased Grade I-IV VIPN in childhood ALL patients (Lopez-Lopez et al., 2016). Zgheib et al. studied *ABCC2* variant rs717620, which was not significantly related to the incidence and severity of VIPN (Zgheib et al., 2018).

In addition to the above-mentioned variants, none of the other SNPs evaluated in *ABCC3* through *ABCC10* were shown to be related to VIPN.

#### 3.2.2 SLC5A7

The *SLC5A7* gene encodes the choline transporter SLC5A7 (Solute Carrier Family 5 Member 7) involved in a distal inherited type of motor neuropathy and congenital muscle weakness syndrome (Barwick et al., 2012; Bauché et al., 2016). ADME analysis identified associations between VIPN and *SLC5A7* SNP variant rs1013940 (g.107992192A > G; *p* = 9.00 × 10^−4^; OR 8.60; 95% CI 1.68–44.15), the most severe Grade III/IV VIPN was experienced by all homozygous risk variant carriers (CC) (Wright et al., 2019).

### 3.3 Genes Related to Pharmacodynamics of VCR

#### 3.3.1 CEP72

The centrosomal protein encoded by the *CEP72* (Centrosomal Protein 72) gene is crucial for the formation of microtubules, and defects in the expression and function of centrosomes may result in improper assembly of the mitotic spindles (Oshimori et al., 2009). SNP variant rs924607 T allele in the *CEP72* promoter can create a transcription suppressor binding site, thereby reducing the expression of *CEP72* in human neurons and leukemia cells, and increasing their sensitivity to VCR. VIPN risk was significantly increased in *CEP72* rs924607 TT genotype carriers (Diouf et al., 2015). The g.609978C > T replacement on rs924607 creates a binding site for the NKX-6.3 transcriptional inhibitor, and molecular models show that the g.609978C > T mutation alters the flexibility of the target DNA duplex and significantly enhances the binding affinity of NKX-6.3 to the T allele. Electrophoresis migration analysis demonstrated that NKX-6.3 binding to the risk allele (T) of *CEP72* was significantly enhanced, which was consistent with the observed low expression of *CEP72* and the risk (T) allele (Stock et al., 2017).

VIPN has shown major axon involvement and is more pronounced in motor neurons (Kavcic et al., 2017). Kavčič et al. (2020) found that VCR treatment caused a significant decrease of the mean distal amplitude of motor neuron potentials in the tibialis nerve in pediatric patients carrying the TT *CEP72* rs924607 genotype compared to those with the CT genotype (*p* = 0.009; 95% CI 1.761–12.931).

Gutierrez-Camino et al. (2016) and Zgheib et al. (2018) reported that there was no association between *CEP72* rs924607 and VIPN. However, Diouf et al. (2015) found that homozygous TT genotype carriers had more severe neuropathy than CC or CT genotype carriers. Similarly, in Wright and others’ study, the *CEP72* rs924607 TT genotype was significantly associated with VIPN (*p* = 0.02; OR 3.43; 90% CI 1.15–10.3) (Wright et al., 2019).

It is important to note that these studies were conducted in populations with different genetic backgrounds. Both studies Diouf et al. (2015) and Wright et al. (2019) that confirmed the association were performed among North American children versus the Saudi population (Zgheib et al., 2018) and the Spanish population (A. Gutierrez-Camino et al., 2016). The study by Gutierrez-Camino and others was the only one to analyze VIPN occurrence during the whole 4 weeks treatment induction period, while others evaluated VIPN at all stages of treatment. Notably, this study also found the VIPN mainly occurred in the later stages of treatment (A. Gutierrez-Camino et al., 2016). A meta-analysis for the Spanish subgroup revealed a significant increased risk of VIPN in childhood ALL patients carrying the *CEP72* rs924607 TT genotype (OR 2.28; *p* = 0.02; 95% CI 1.16–6.87) (Zečkanović et al., 2020).

#### 3.3.2 TUBB

*TUBB* (Beta Tubulin) genes encoding β-tubulin include *TUBB1*, *TUBB2A*, *TUBB2B*, *TUBB3*, and *TUBB4*. The related pathways include neurodegenerative pathways, which are involved in a variety of diseases, cell cycles, and mitosis. According to Kavallaris (Kavallaris, 2010), VIPN is caused by VCR binding to the subunits of the α/β tubulin heterodimer and inhibiting microtubule polymerization. Therefore, studying the genetic variations of these genes may help to understand the occurrence and development of VIPN. However, limited studies have shown that the genetic variations of this gene have nothing to do with VIPN (Ceppi et al., 2014; Martin-Guerrero et al., 2019).

#### 3.3.3 MAPs

VCR damages the microtubules in the axonal part of the nerves and leads to impaired axonal transport and loss of nerve function, resulting in polyneuropathy. Microtubule-associated proteins (MAPs) promote microtubule assembly and stability in neuronal axonal chambers. Theoretically, low expression of MAPs may decrease microtubule stability and thus increase the incidence and severity of multiple neuropathy (N. M. Verrills et al., 2003). MAP4 can stabilize the mitotic spindle to slow down the polymerization of microtubules. However, several studies (Hartman et al., 2010; Ceppi et al., 2014; Skiles et al., 2018; Martin-Guerrero et al., 2019) have shown that the polymorphism of *MAPs* did not affect sports performance in childhood ALL patients treated with VCR. Genetic variation in the *MAPT* (Microtubule Associated Protein Tau) (rs11867549) gene was not associated with VIPN (Hartman et al., 2010; Skiles et al., 2018; Martin-Guerrero et al., 2019).

#### 3.3.4 ACTG1

ACTG1 (Actin Gamma 1) is associated with deafness, autosomal dominant inheritance, and Baraitser-Winter syndrome. ACTG, encoded by the *ACTG1* gene, is the major cytoskeleton protein (Ceppi et al., 2014). *ACTG1* variation is expected to affect the exon splicing regulatory sequence (Zhao et al., 2008). The *ACTG1* genetic variant rs1135989 was significantly correlated with high-grade VIPN, which successfully predicted the VIPN risk in a validation cohort, and the predicted OR value was almost the same as the observed OR value (Abaji et al., 2018). Patients carrying the A allele with the synonymous g.81510981G > C/A (rs1135989) variant in the *ACTG1* gene had a higher risk of Grade III/IV neurotoxicity compared with other genotype carriers (OR 2.8; 95% CI 1.3–6.3; *p* = 0.008). Patients carrying the A allele had more frequent episodes of Grade III/IV neurotoxicity (*p* = 0.008) and a lower tolerated VCR dose (*p* = 0.02) than patients without the A allele (Ceppi et al., 2014). In all recurrent cases, *ACTG1* mRNA levels decreased, and *ACTG1* mutations were also detected in VCR-resistant leukemia cell lines (Verrills et al., 2006b).

#### 3.3.5 CAPG

The *CAPG* (Capping Actin Protein) gene encodes a member of the Gelsolin/Villin family of Actin regulatory proteins. The encoded protein reliably blocks the barbed ends of the F-actin filaments in a manner regulated by Ca^2+^ and phosphoinositide, but does not cut off the formed actin filaments. By covering the prickly ends of actin filaments, coding proteins help to control actin-based movement in non-muscle cells (Shekhar et al., 2016). Higher-level neurotoxicity was also associated with a SNP variant rs2229668 (g.85401860C > T/G) in the *CAPG* gene. *CAPG* mediates crosstalk between actin and the microtubule cytoskeleton (Verrills et al., 2006a; Hubert et al., 2009). *CAPG* sequences prior to transcription start sites might affect mRNA regulation (Fairbrother et al., 2002). The neurotoxicity risk increased as the number of alleles increased (OR 2.1; 95% CI 1.1–3.7; *p* = 0.02). The SNP variant rs3770102 (g.85410714G > T/A/C) in the *CAPG* gene, located at 17 nucleotides upstream of the transcription start site, had a protective effect on high-grade neurotoxicity, AA genotype carriers had a lower risk of toxicity (OR 0.1; 95% CI 0.01–0.8; *p* = 0.009) and the frequency of toxic episodes was lower (*p* = 0.007) (Ceppi et al., 2014).

### 3.4 Other Genes

#### 3.4.1 SYNE2

The *SYNE2* (Spectrin Repeat Containing Nuclear Envelope Protein 2) gene is located on human chromosome 14q23, and encodes a protein that binds cytoplasmic F-actin. This binding tethers the nucleus to the cytoskeleton and helps the nucleus maintain its structural integrity. The *SYNE* gene also encodes a new class of proteins that contain Spectrinrepeat (SR), also known as Nesprin proteins, including isoform NESPRIN-2 (Rajgor et al., 2012). This protein binds to the multifunctional actin in the cytoplasm and is an important cytoskeleton protein that maintains the nuclear membrane structure and cell structure. It is widely distributed in various tissues (Wilson and Holzbaur, 2015). It is associated with neurological diseases, axonal neuropathy in several cases (Madej-Pilarczyk et al., 2015) and plays a key role in neurogenesis and neuronal migration (Zhang et al., 2009; Rajgor and Shanahan, 2013; Cartwright and Karakesisoglou, 2014). *SYNE2 rs2781377* minor allele carriers have an increased risk of developing VIPN, which was proportional to the copy number of the risk A allele (OR 2.5; 95% CI 1.2–5.2; *p* = 0.01) (Abaji et al., 2018).

#### 3.4.2 BAHD1

BAHD1 (Bromo adjacent homology domain containing 1) is a protein encoded by the *BAHD1* gene in humans. *BAHD1* is an important regulator of gene silencing during the formation of heterochromatin and plays an important role in inhibiting proliferation and survival genes or as a regulator of inflammation through the TNF signaling pathway (Zhu et al., 2015). Abnormal epigenetic characteristics of *BAHD1* in controlling the spatial structure of the genome might be the cause of many diseases, and these abnormal-epigenetic characteristics were associated with sensory and autonomic neuropathy (Lakisic et al., 2016; Libertini et al., 2015; Z.; Sun et al., 2014). Recently, the SNP variant rs3803357 (g.40459356C > G/A) in the *BAHD1* gene was found to be associated with a lower incidence of toxicity (OR 0.35; 95% CI 0.2–0.7; *p* = 0.007). Therefore, the minor A allele of rs3803357 had a protective role against high-grade VIPN (Abaji et al., 2018).

#### 3.4.3 COCH

The cochlin protein is encoded by the *COCH* gene in humans. The mutation of rs1045644 in the *COCH* gene might be related to the neuropathy caused by VCR (L. Li et al., 2019), but is not supported by other studies (Sawaki et al., 2020). Li et al. found that the SNP variant rs1045466, located on chromosome 14 and part of the *COCH* gene, was associated with lower neuropathy scores in childhood ALL patients, probably due to the overexpressed cochlin protein facilitating recovery from the toxicity of VCR (L. Li et al., 2019).

#### 3.4.4 MRPL47

Mitochondrial ribosomal proteins (MRPs) are essential components for the structural and functional integrity of the mitoribosome complex. Mutations in MRPs family of genes may be associated with neurological diseases, muscle diseases, and developmental disorders due to their ability to reduce ATP production (O’Brien, 2002). *MRPL47* gene localizes to areas associated with sensory nerve disease, indicating a potential genetic susceptibility to VCR neurotoxicity (Kenmochi et al., 2001; O’Brien, 2002). A common variant with a reference number rs10513762 (g.179588987C > T) in the *MRPL47* gene was found to be significantly associated with an increased risk of Grade III/IV VIPN on univariate analysis (OR, 3.3; 95% CI, 1.4–7.7; *p* = 0.01), and after controlling for multiple comparisons (OR, 3.9; 95% CI, 1.5–10; *p* = 0.004) (Abaji et al., 2018). Thus, genetic variants in *MRPL47* genes might be considered as putative new risk factors for VIPN in childhood ALL patients.

#### 3.4.5 ITPA

The *ITPA* gene, located on chromosome 20, encodes an inosine triphosphate pyrophosphohydrolase (ITPase). The enzyme ITPase hydrolyzes inosine triphosphate and deoxyinosine triphosphate into monophosphate nucleotide and diphosphate. In one study, Franca et al. found that four patients carrying the *ITPA* SNP rs1127354 (g.3213196C > G/A) homozygous mutation had a 13-fold increased risk of severe neurotoxicity compared with wild-type patients (OR 13.23; 95% CI 1.74–100.65; *p* = 0.013) (Franca et al., 2017). In the AIEOP-BFM 2000 protocol, *ITPA* gene variation was associated with grade III/IV neurotoxicity during the remission induction phase (OR 13.23; 95% CI 1.74–100.65) (Franca et al., 2017). However, this result is not certain to be related to VCR toxicity because the observed neurotoxicity is not clearly caused by VCR alone. Homozygotes for the g.3213196C > G/A missense mutation had zero erythrocyte ITPase activity, whereas g.3213196C > G/A heterozygotes averaged 22.5% of the control mean, a level of activity consistent with impaired subunit association of a dimeric enzyme (Sumi et al., 2002).

#### 3.4.6 DPYD

The protein Dihydropyrimidine Dehydrogenase (DYPD) encoded by the *DPYD* gene is a pyrimidine catabolic enzyme and the initial and rate-limiting factor in the pathway of uracil and thymidine catabolism. Mutations in this gene result in DYPD deficiency, an error in pyrimidine metabolism associated with thymine-uraciluria, and an increased risk of toxicity in cancer patients after receiving 5-fluorouracil chemotherapy. Studies have found that the intron SNP rs1413239 in the *DPYD* gene is related to delayed peripheral neuropathy caused by VCR. This genetic polymorphism can increase the risk and severity of delayed peripheral neuropathy (OR 3.29; 95% CI 1.47–7.37; *p* = 5.40 × 10^−3^) (Broyl et al., 2010).

#### 3.4.7 RALBP1

*RALBP1* (Rala Binding Protein 1) is a protein-coding gene. It has been linked to lung cancer. The low affinity, high volume transporter RALBP1 has similar but different substrate specificity to *ABCC1* and *ABCG2* (Awasthi et al., 2005b). RALBP1 induces a stress response that protects ATP-dependent GS-E and xenotoxins, such as chemotherapy transporters (Singhal et al., 2006). Drake and others found for the first time that overexpression of *RALBP1* resulted in an extensively drug-resistant phenotype in human erythemaemia cells K562 (Drake et al., 2007). Multidrug resistance is caused by overexpression of *RALBP1*. The effect of *RALBP1* transfection on VCR was detected by a cytotoxicity assay. Half inhibition concentrations in wild-type, empty vector transfected and Ralbp1 transfected cells were determined in cells that grew 24 h after the addition of IgG or anti-*RALBP1* immunoglobulin G. While empty vector transfection had little or no effect, the overexpression of *RALBP1* was 2–5 times more resistant to VCR. Drake et al. (2007) found that anti-RALBP1 IgG reduced the semi-inhibitory concentration in all cell lines and caused the resistance of *RALBP1* overexpressed cells to be reversed to wild-type or empty vector transfected cells. So far, only one study has studied the changes in VCR in humans. When only patients with high toxicity (Grade III/IV) were considered, a significant association was found between one haplotype of *RALBP1* and neurotoxicity. However, after FDR correction, the statistical significance could not be retained (Lopez-Lopez et al., 2016).

#### 3.4.8 TTPA

*TTPA* (Alpha Tocopherol Transfer Protein) is a protein-coding gene. *TTPA* mutations can lead to vitamin E deficiency alone, leading to ataxia (Euch-Fayache et al., 2014; Schmölz et al., 2016). A meta-analysis found that one variant associated with *TTPA* (rs10504361; *p* = 6.85 × 10^−4^, OR 1.98; 95% CI 1.34–2.94) might be associated with hereditary neuropathy (Wright et al., 2019). Wright et al. evaluated the difference in the predicted *TTPA* gene expression in the tibial nerve using S-PrediXcan in the GWAS-Array genotyped cohort, and the analysis showed that the expression was predicted to be higher in VIPN cases than in the control group (*p* = 6.1 × 10^−5^) (Wright et al., 2019). Levels of *TTPA* in the tibial nerve were elevated in some cases, and sensory conduction in the tibial nerve has been shown to be impaired in all VCR-treated patients (Vainionpää et al., 1995). The tibial nerve has a motor function by supplying the leg muscles, including the tibialis posterus, which is involved in plantar flexion (Rosson et al., 2009). This suggests that the tibial nerve may be a suitable model for studying the expression of VIPN-related genes.

#### 3.4.9 Genomics of MicroRNAs

MicroRNAs (miRNAs) are approximately 22 nucleotides long and are small noncoding RNAs that function in posttranscriptional gene silencing. Genetic variations in miRNA genomics can alter miRNA levels or function, which may affect the expression of their target genes.

The rs7896283 CC genotype in *miR-4481* studied by Gutierrez-Camino et al. was related to VIPN, which was involved in the regulation of axon guidance pathway genes (Á. Gutierrez-Camino et al., 2018). The C allele of rs7896283 was located in the pre-miRNA sequence, and the T allele replaced the C allele, causing negative energy changes. This replacement changes the miRNA from an unstable state to a stable state (ΔG = −1.2 kcal/mol). The increase in structural stability of pre-miRNA might enhance the product of mature miRNA (Gong et al., 2012), thereby leading to a decrease in its target protein expression. Both the KEGG and REACTOME databases predict that axon guidance is the most important targeting pathway for *miR-4481*. When a peripheral nerve is damaged, axonal guidance participates in its spontaneous regeneration (Chiono and Tonda-Turo, 2015). Therefore, the C allele of rs7896283 might increase the stability of *miR-4481*, resulting in reduced expression of its target genes. Therefore, the low expression of genes involved in peripheral nerve regeneration may be responsible for the increase in peripheral neuropathy. rs7896283 was associated with a 2.6-fold increased risk of neurotoxicity (*p* = 0.017), and the C allele was associated with high-grade neurotoxicity (*p* = 0.0148). However, no significant *p* value could be retained after FDR correction (Á. Gutierrez-Camino et al., 2018).

In Gutierrez-Camino’s study, it was found that SNP variant rs12402181 in *miR-3117* could reduce the toxicity of VIPN (Á. Gutierrez-Camino et al., 2018). AG/AA genotypes were associated with a 0.16-fold reduction in risk of Grade I-IV neurotoxicity (*p* = 0.00042), whereas AA genotypes were never found in patients with peripheral neurotoxicity. The SNP was located in the seed region of *miR-3117-3p*, so the allele g.66628488G > T/A would affect the accurate recognition of its target mRNA sequence. *MiR-377-3p* targets VCR transporters, such as ABCC1 and RALBP1. The *ABCC1* gene encodes MRP1, which is involved in VCR transport (Winter et al., 2013). RALBP1, a highly active protein, is also involved in the process of VCR removal (Awasthi et al., 2005a). Therefore, SNP variant rs12402181 can affect *ABCC1* and *RALBP1*, and then increase their expression. High expression of *ABCC1* and *RALBP1* genes may lead to increased efflux of VCR from cells, reducing the risk of VIPN. Significant correlations were also found in low and high toxicity grade analyses during the induction phase of treatment, with *p* values of 0.0069 and 0.0099, respectively (Á. Gutierrez-Camino et al., 2018). After FDR correction, however, the *p* value was not significant (*p* = 0.06).

Genetic polymorphisms in *microRNA-6076* (Á. Gutierrez-Camino et al., 2018), *microRNA 202* (Martin-Guerrero et al., 2019), have been found to be associated with VIPN, but the statistical significance could not be retained after FDR correction. Further studies are needed to validate their findings.

## 4 Future Perspective

As therapies begin to prolong life in the majority of cancer patients, drug-related toxicity takes on a greater degree of importance in cancer care. Avoidance of acute and chronic adverse drug reactions can aid choices among apparently equal treatment options. This is pretty relevant in light of effective therapies, as active therapies can be compromised due to debilitating toxic effects, especially neurotoxicity, such as VIPN. The long-term or final goal of pharmacogenomic studies is to transform findings with regard to the genetic basis of drug responses into more effective and less toxic treatments for individual patients.

SNP variants in the *CEP72* gene have a great potential to become biomarkers in clinical practice, because of this well-performed study by Diouf et al. (2015) containing many key elements, such as genome-wide discovery in patients from well-conducted clinical trials, replication in a multicenter cohort, statistical robustness, and laboratory correlative findings that contribute to biologic plausibility (McLeod, 2015).

As well known, VIPN is a dose-limiting side effect of VCR treatment in childhood ALL patients, leading to diminished quality of life. The good way of implementing personalized dosing is by combining pharmacogenomic test with blood drug levels measurement (Pacanowski and Liu, 2020), called therapeutic drug monitoring (TDM). So far, nothing is known about the association between VIPN and plasma VCR concentrations. Little is known about the relationship between VCR dose and system exposure. Perhaps, TDM research can help us better understand VCR and VIPN from another novel perspective.

The aforementioned various pharmacogenomic studies have been reported with the goal of better understanding of human genetic variability and its influence on VIPN and many genetic polymorphisms have been individually studied. As high-throughput technologies provide us with increasingly high-quality data on the genome and transcriptome properties of childhood ALL patients and their response to treatment, we will surely discover reliable VIPN predictors. However, predictions by using single genetic factor are full of challenges. Because there are many confounding factors, such as disease phenotype, comorbidity, age, developmental degrees of organs, race and ethnicity. Therefore, combination of genetic, environmental, and personal variables may facilitate understanding VIPN mechanisms thereby predicting and improving the side effects of VCR.

In the future, we hope to see more functional studies and have reliable VIPN biomarkers in the clinical environment, which will help us better understand the mechanism of VIPN with aims to ultimately prevent it, improve the effectiveness of patient treatment, reduce adverse reactions and improve the short-term along with long-term quality of life in cancer patients. Further well-designed researches jointing the efforts of researchers, clinicians and patients are highly needed.

## Glossary

ABC: ATP-binding cassette transporter
ABCB1: ATP-binding cassette subfamily B member 1
ABCB4: ATP-binding cassette subfamily B member 4
ABCC1: ATP-binding cassette subfamily C member 1
ABCC2: ATP-binding cassette subfamily C member 2
ACTG1: actin gamma 1
ALL: acute lymphoblastic leukemia
BAHD1: bromo adjacent homology domain containing 1
CAPG: capping actin protein
Cavs: voltage-gated calcium channels
CCCG-ALL-2015: China children’s cancer group ALL-2015 protocol
CD8^+^: cytotoxic T cells
CEP72: centrosomal protein 72
CI: confidence interval
cMOAT: canalicular multispecific organic anion transporter 1
CTX: cyclophosphamide
COCH: cochlin
COS: cyclooxygenase
DRG: dorsal root ganglion
DNR: daunorubicin
DXM: dexamethasone
DPYD: dihydropyrimidine dehydrogenase
EFS: event free survival
ETAA1: activator of ATR kinase
FDR: false discovery rate
ITPA: inosine triphosphate pyrophosphatase
Kv: voltage-gated potassium
L-asp: L-asparaginase
MAPs: microtubule-associated proteins
MAPT: microtubule-associated protein tau
MAP4: microtubule-associated protein 4
MDR: multidrug resistance
miRNAs: microRNAs
miR-202: microRNA 202
MRP: multidrug resistance-associated protein
MRP1: multidrug resistance-associated protein 1
MRP2: multidrug resistance-associated protein 2
MRPs: mitochondrial ribosomal proteins
MTNR1B: melatonin receptor 1B
Navs: voltage-gated sodium channels
NADPH: nicotinamide adenine dinucleotide phosphate
NOS: nitric oxide synthase
OS: overall survival
PD: pharmacodynamics
PDN: prednisone
P-gp: P-glycoprotein
PK: pharmacokinetic
RALBP1: rala binding protein 1
ROS: reactive oxygen species
SLC5A7: solute carrier family five member 7
SNPs: single-nucleotide polymorphisms
SNVs: single nucleotide variants
SR: spectrinrepeat
SYNE2: spectrin repeat containing nuclear envelope protein 2
TRP: transient receptor potential
TTPA: alpha tocopherol transfer protein
TUBB: beta tubulin
VCR: vincristine
VIN: vincristine-induced neuropathy
VIPN: vincristine induced peripheral neuropathy
